# 三氟乙酸调控合成缺陷型UiO-66用于高效去除全氟壬烯氧基苯磺酸钠

**DOI:** 10.3724/SP.J.1123.2026.01019

**Published:** 2026-10-08

**Authors:** Xuan WANG, Chunxiao WANG, Yan XIA

**Affiliations:** 南开大学化学学院分析科学研究中心，天津市生物传感与分子识别重点实验室，天津 300071; Research Center for Analytical Science，College of Chemistry，Nankai University，Tianjin Key Laboratory of Biosensing and Molecular Recognition，Tianjin 300071，China

**Keywords:** 金属有机框架, 全氟壬烯氧基苯磺酸钠, 缺陷工程, 吸附, 氟-氟相互作用, metal-organic frameworks, sodium *p*-perfluorous nonenoxybenzenesulfonate, defect engineering, adsorption, fluorine-fluorine interaction

## Abstract

全氟壬烯氧基苯磺酸钠（OBS）作为全氟辛烷磺酸（PFOS）的替代品，具有更强的生物积累性、难降解性和细胞毒性。本研究提出了一种缺陷工程策略，通过溶剂热法合成三氟乙酸（TFA）调控的UiO-66系列材料（U-TFA-10 M和UN-TFA-10 M等）用于吸附去除OBS。扫描电子显微镜（SEM）、透射电子显微镜（TEM）、X射线衍射（XRD）结果表明，U-TFA-10 M和UN-TFA-10 M并未呈现规则的八面体形貌，具有丰富的结构缺陷。傅里叶变换红外光谱（FT-IR）、X射线光电子能谱（XPS）等进一步证实材料中成功引入三氟甲基（-CF_3_）。优化结果表明，在溶液pH为3时，U-TFA-10 M和UN-TFA-10 M对OBS的吸附效果最佳，离子强度的影响可忽略不计。吸附等温线和吸附动力学模拟结果表明，吸附过程符合Langmuir模型和伪二级动力学模型，用Langmuir模型计算的饱和吸附容量分别为684.9 mg/g和602.4 mg/g。同时U-TFA-10 M和UN-TFA-10 M展现出良好的再生能力，在吸附-解吸-再生过程后仍能保持较高的吸附效率。机理研究表明，其高效吸附性能源于多种吸附机制的协同作用：孔径匹配、静电相互作用、酸碱相互作用、氟-氟相互作用和疏水相互作用。通过与已报道的吸附剂相比，U-TFA-10 M和UN-TFA-10 M对OBS兼具较高的吸附容量和快速吸附的显著优势，是一种有效且易制备的固体吸附剂，在污染物吸附分离的应用中具有较大的潜力。

## Abstract (en)

Per- and polyfluoroalkyl substances （PFAS）， widely known as “forever chemicals”， present a severe and escalating global threat to ecological systems and human health. Their extreme persistence originates from remarkably strong carbon-fluorine bonds， while their amphiphilic nature facilitates widespread environmental dispersal and bioaccumulation. Among them， sodium *p*-perfluorous nonenoxybenzenesulfonate （OBS） has been extensively used as a commercial alternative to perfluorooctanesulfonic acid （PFOS）， which is under strict global regulation. However， mounting evidence has revealed that OBS exhibits higher bioaccumulation， higher environmental persistence， and significant cytotoxicity， raising urgent environmental concerns and creating an urgent demand for efficient remediation technologies. To tackle this challenge， this study proposes a defect-engineering strategy for the design of advanced adsorbent materials. Specifically， a series of trifluoroacetic acid （TFA）-modulated UiO-66-type metal-organic frameworks （MOFs）， denoted as U-TFA-10 M， UN-TFA-10 M etc.， were successfully synthesized via a solvothermal method. The intentional introduction of structural defects was designed to increase the density of active sites and tailor surface properties， thereby achieving enhanced pollutant capture performance. Comprehensive characterization confirmed the successful material synthesis and modification. Scanning electron microscopy （SEM） and transmission electron microscopy （TEM） images revealed that U-TFA-10 M and UN-TFA-10 M particles exhibited irregular， fragmented morphologies， a stark contrast to the regular octahedral shape of pristine UiO-66， visually indicating abundant structural defects. X-ray diffraction （XRD） patterns displayed a new broad peak in the 2*θ* range of 3°-5°， further confirming defect formation. Fourier-transform infrared spectroscopy （FT-IR） identified characteristic vibrations： peaks at 556 cm^–1^ and 482 cm^–1^ corresponded to Zr-O coordination， while a distinct peak at 1 212 cm^–1^ confirmed the presence of -CF_3_ groups. X-ray photoelectron spectroscopy （XPS） provided definitive evidence， with a clear F 1*s* signal verifying the successful incorporation of trifluoromethyl moieties into the MOF framework—a critical modification for enabling specific interactions with the target pollutant. Systematic adsorption evaluation and optimization were conducted. The optimal synthesis condition was identified as using 10 mol/L TFA as the modulator. The pH of the solution profoundly influenced adsorption， with maximum OBS removal achieved at pH 3， where the protonated adsorbent surface exerted strong electrostatic attraction towards anionic OBS molecules. Notably， adsorption efficiency remained stable across a range of ionic strengths （Cl^–^ mass concentration 0-100 mg/L）， indicating strong resistance to interference from common salts， a vital advantage for practical application. Adsorption equilibrium data fitted the Langmuir isotherm model （*R* ^2^ of 0.999 9 for U-TFA-10 M and 0.999 5 for UN-TFA-10 M）， suggesting monolayer adsorption on homogeneous active sites. The maximum adsorption capacities were 684.9 mg/g for U-TFA-10 M and 602.4 mg/g for UN-TFA-10 M. Kinetic analysis followed the pseudo-second-order model （*R* ^2^ of 0.999 9 for U-TFA-10 M and 0.998 8 for UN-TFA-10 M）， implying chemisorption as the rate-limiting step. The materials also exhibited excellent regenerability and reusability， maintaining high efficiency after repeated adsorption-desorption cycles. Mechanistic studies revealed that the superior performance stems from a synergistic combination of multiple interactions： （1） pore-size matching for physical entrapment； （2） electrostatic attraction between the protonated surface and the OBS sulfonate group； （3） acid-base interaction between unsaturated metal sites （Lewis acids） and the sulfonate group； （4） specific fluorine-fluorine （F-F） interaction between the -CF_3_ groups and the OBS perfluorinated chain， enhancing selectivity； and （5） hydrophobic interaction promoting partitioning from the aqueous phase. Compared with other reported OBS adsorbents， U-TFA-10 M and UN-TFA-10 M combined ultrahigh capacity and fast kinetics， with a simple and scalable synthesis route， showing great practical potential. In summary， this work successfully developed U-TFA-10 M and UN-TFA-10 M as efficient， stable， and easily prepared adsorbents for OBS removal. It also validated defect engineering coupled with -CF_3_ functionalization as a powerful material design strategy. The mechanistic insights provide a valuable theoretical foundation for developing next-generation materials for the separation and remediation of persistent aquatic pollutants.

全氟和多氟烷基化合物（per- and polyfluoroalkyl substances， PFAS）是一类人工合成的有机化合物，由于其高度的疏水性、憎油性、热稳定性和化学稳定性，广泛应用于纺织品、不粘锅和消防泡沫等多个领域^［1］^。在众多的PFAS中，全氟辛酸（perfluorooctanoic acid， PFOA）和全氟辛烷磺酸（perfluorooctanesulfonic acid， PFOS）是最典型的两种^［2］^。PFOS具有稳定的C-F键（115 kcal/mol，相当于481 kJ/mol）^［3］^，在水、土壤和生物体中具有高度的累积性和持久性^［4］^，对生态系统和人类健康造成极大威胁。由于PFOS已被许多国家和地区禁用或者有严格的限制，全氟壬烯氧基苯磺酸钠（sodium *p*-perfluorous nonenoxybenzenesulfonate， OBS）凭借价格低廉、合成简单的优点成为PFOS的替代品。

作为一种新型PFAS，OBS在C-F链上有一个醚基和一个芳基，自然降解更加困难^［5］^。由于OBS疏水和亲水的双重性质，它具有比PFOS更强的生物积累性^［4］^。调查表明，中国的OBS产能达3 500吨/年^［4］^，在大庆油田周围的水样中检测出OBS残留，最高浓度达3 200 ng/L^［6，7］^，2020年山东省小清河流域地表水中检测到的OBS残留量已经超过了PFOS^［8］^。已有研究表明，OBS对斑马鱼和小鼠可产生各种不良影响，包括肠道屏障功能障碍、肝脏代谢异常、昼夜节律改变和甲状腺功能障碍等^［9］^，在人肝癌细胞中表现出比PFOS更严重的细胞毒性^［4］^。因此，开发高效的OBS去除方法，对维护生态系统和保障人类健康具有现实紧迫性和重要意义。

目前，PFAS的检测方法主要有液相色谱-质谱法（LC-MS）^［10-13］^和气相色谱-质谱法（GC-MS）^［14］^。然而，由于环境样品中PFAS的水平较低，样品前处理是非常必要的。去除PFAS的方法主要有生物降解^［15］^、光催化^［16］^、膜分离^［17］^、混凝^［18］^、吸附法^［19］^，其中生物降解所需时间长并且去除不够完全，光催化技术易产生有毒中间体或副产物且成本高，膜分离法膜面易污染，混凝法具有不可控性，而吸附法操作简单、去除效果好，是目前最可行、最经济的去除和预处理方法。传统的吸附剂如活性炭、沸石、离子交换树脂、碳纳米管以及分子印迹材料等已经被用作去除PFAS，其吸附机理主要为PFAS与吸附剂上相应位置的特殊相互作用和疏水效应^［20］^，然而这些吸附剂的稳定性、选择性和可再生能力较差，导致吸附效率低下。因此，开发一种孔径合适、活性位点丰富、稳定性好的吸附剂是提高吸附效果的关键。

金属有机框架材料（MOFs）是由金属离子或簇与有机配体自组装而成的一类多孔材料，由于制备简单、孔径可调节、结构可设计、比表面积大等优点，在传感^［21］^、催化^［22］^、储能^［23］^和样品前处理^［24］^等多个领域得到了广泛的应用。然而，受稳定性的限制，目前用于样品前处理的MOFs仍然只有少数几类（如MIL系列和ZIF系列等）。因此，根据目标物的特性来精准设计或功能化MOFs吸附剂并将其应用于样品前处理领域具有重要意义^［25］^。近年来的研究证实了氟-氟相互作用的存在，即C-F…F-C^［26］^。含氟官能化吸附剂因其能选择性地从复杂基质中吸附PFAS而备受关注^［20，27］^。其中，氟化MOFs抗水性强、稳定性强。UiO系列MOFs因其易于氟化而备受关注。例如，Jiang等^［28］^采用水热法制备了氟化金属有机框架材料（UiO-66-F_4_），对OBS表现出高效选择性吸附（最大吸附量约451 mg/g），其机理主要依赖于疏水作用和氟-氟相互作用。此外，通过调控UiO-66中缺陷的类型（如缺失连接体缺陷、缺失节点缺陷）与浓度，可显著提高其吸附效率。例如，在UiO-66中引入连接体缺陷已被证明可以增强对全氟正丁烷磺酸（perfluorobutanesulfonic acid， PFBS）和全氟辛烷磺酸（PFOS）的吸附^［29］^；Cheng等^［30］^通过低共熔溶剂控制UiO-66的尺寸和缺陷实现全氟烷基化合物的有效吸附。

本研究使用三氟乙酸（TFA）对UiO-66以及UiO-66-NH_2_进行缺陷调控，合成了U-TFA-10 M和UN-TFA-10 M并用于OBS的高效、选择性吸附（图1）。TFA作为一种强酸性有机调节剂，可以诱导UiO-66系列材料产生结构缺陷并且引入三氟甲基（-CF_3_），从而改变材料的孔隙结构和表面性质，通过氟-氟相互作用和疏水作用增强对OBS的吸附能力。此外，UiO-66-NH_2_中氨基的引入还能为进行合成后修饰提供锚定位点，为进一步开发更高性能的吸附材料奠定基础。

**图1:**
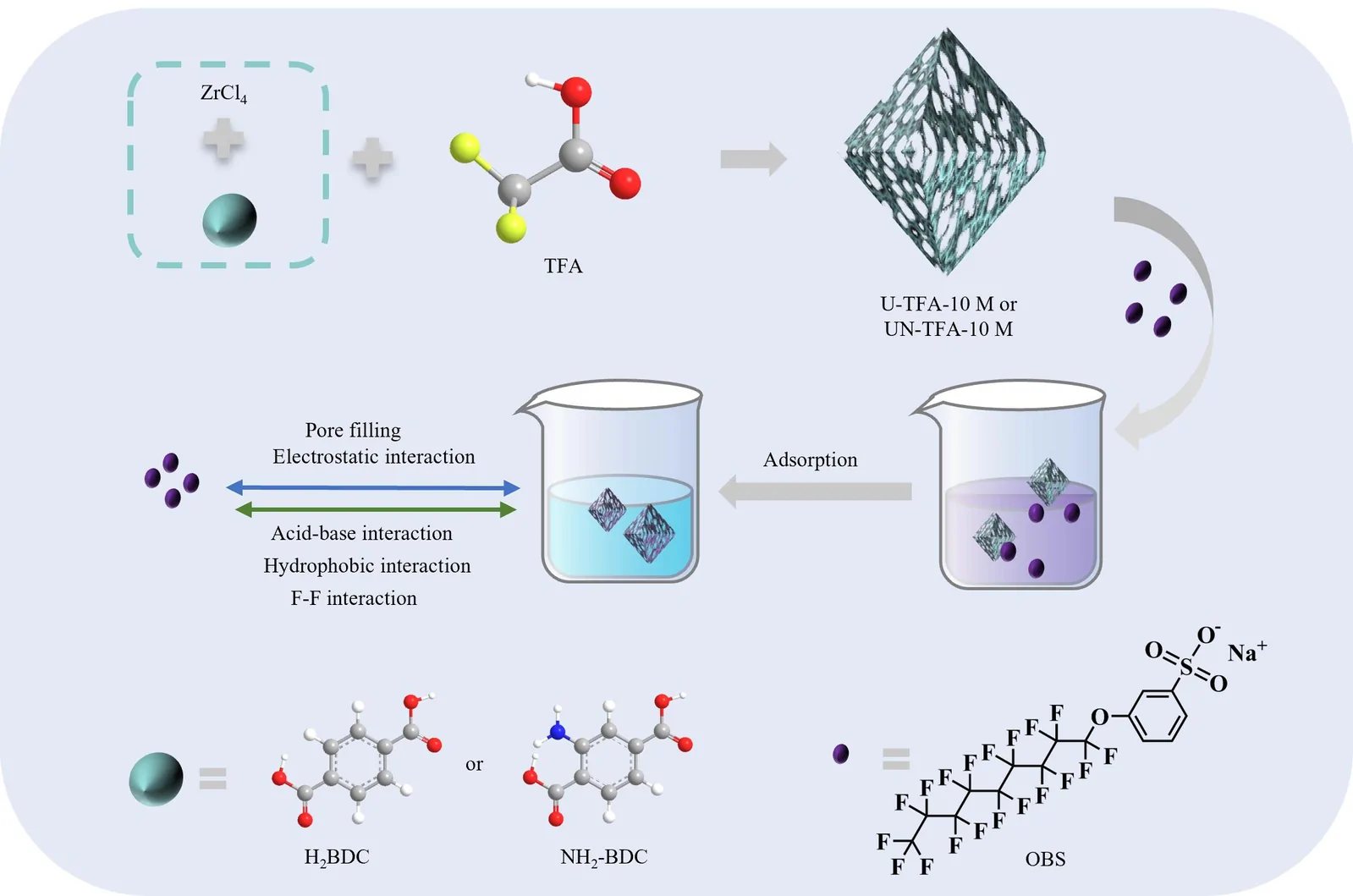
TFA调控合成UiO-66系列材料以及吸附OBS的流程示意图 TFA： trifluoroacetic acid； U-TFA-10 M： TFA-modulated UiO-66； UN-TFA-10 M： TFA-modulated UiO-66-NH_2_； H_2_BDC： 1，4-dicarboxybenzene； NH_2_-BDC： 2-aminobenzene-1，4-dicarboxylic acid； OBS： sodium *p*-perfluorous nonenoxybenzenesulfonate.

## 1 实验部分

### 1.1 仪器与试剂

SmartLab X射线衍射仪（XRD，日本Rigaku公司）；Nicolet iS50傅里叶变换红外光谱仪（FT-IR，美国Thermo Fisher公司）；Apreo S LoVac扫描电子显微镜（SEM，美国FEI公司）；HT7800透射电子显微镜（TEM，日本Hitachi公司）；ESCALAB 250Xi X-射线光电子能谱仪（XPS，美国Thermo Fisher公司）；UV-3600紫外-可见分光光度计（UV-Vis，日本Shimadzu公司）；纳米粒度分析仪（Zetasizer Nano ZS，英国Malvern Panalytical公司）；冷冻恒温摇床（ATS-032R，上海Chemstar Ltd.公司）；ASAP 2460氮气吸-脱附测定仪（美国Micromeritics公司）。

氯化锆（ZrCl_4_）、对苯二甲酸（H_2_BDC）、TFA、十二烷基苯磺酸钠（SDBS）、Amberlite^®^ IRA-900阴离子交换树脂（IRA-900）购自上海阿拉丁生化科技股份有限公司；2-氨基对苯二甲酸（NH_2_-BDC）、丙酸（PA）购自梯希爱（上海）化成工业发展有限公司；2-氟苯甲酸（2-FBA）购自上海笛柏生物科技有限公司；苯甲酸（BA）购自上海麦克林生化科技有限公司；OBS购自上海泰坦科技股份有限公司；氯化钠（NaCl）、活性炭购自天津希恩思奥普德科技有限公司；氢氧化钠（NaOH）购自天津市光复精细化工研究所；盐酸（HCl）购自天津市化学试剂六厂；*N，N*-二甲基甲酰胺（DMF）、无水乙醇（EtOH）、甲醇（MeOH）、冰乙酸（AA）购自天津康科德科技有限公司。实验室用高纯水购自天津西青区永清源蒸馏水经营部。

### 1.2 实验方法

#### 1.2.1 UiO-66、UiO-66-NH_2_的合成

通过溶剂热法合成UiO-66、UiO-66-NH_2_。称取ZrCl_4_（0.25 mmol）与H_2_BDC（0.25 mmol）溶于12.5 mL DMF中，转移至反应釜内胆中，超声20 min，将反应釜置于烘箱中，120 ℃加热48 h。反应结束后冷却至室温，离心收集沉淀，用DMF、无水乙醇分别洗涤3次，将反应物置于真空干燥箱中60 ℃干燥24 h。UiO-66-NH_2_合成步骤与之相同，用NH_2_-BDC（0.25 mmol）代替H_2_BDC。

#### 1.2.2 TFA调控UiO-66系列材料的合成

UiO-66-TFA-*n* M（以下简称U-TFA-*n* M，*n* M表示TFA的浓度，其中M代表mol/L）的合成步骤如下：将ZrCl_4_（0.25 mmol）与H_2_BDC（0.25 mmol）用12.5 mL的DMF超声溶解，得到前驱体溶液，转移至反应釜内胆；将8 mmol的TFA用不同量的高纯水分别配制为5、7.5、10、12.5 mol/L的溶液；将不同浓度的TFA溶液加入到前驱体溶液中，超声20 min使其混合均匀；将反应釜置于烘箱中，120 ℃加热48 h。其余步骤同上。UiO-66-NH_2_-TFA-*n* M（以下简称UN-TFA-*n* M）合成步骤与之相同，用NH_2_-BDC（0.25 mmol）代替H_2_BDC。为了进行对比实验，合成了其他单羧酸调控的UiO-66-*X*-10 M（简称U-*X*-10 M，*X*=AA或PA）和UiO-66-NH_2_-*X*-10 M（以下简称UN-*X*-10 M，*X*=AA或PA）。

#### 1.2.3 UiO-66系列材料对OBS的吸附实验

称取400 mg OBS用高纯水超声溶解并定容至1 000 mL得到OBS标准溶液（400 mg/L）。称取2 mg UiO-66、UiO-66-NH_2_，2 mg经不同浓度TFA调节的U-TFA-*n* M、UN-TFA-*n* M以及2 mg使用10 mol/L不同种类调节剂调节的吸附剂（U-*X*-10 M、UN-*X*-10 M，*X*=AA或PA）分别置于7 mL离心管中，加入5 mL OBS标准溶液，将其置于25 ℃、220 r/min摇床吸附12 h，吸附完成后用0.22 μm滤膜过滤，收集上清液进行UV-Vis检测，然后根据OBS的浓度标准曲线计算吸附后浓度，并通过公式（1）计算吸附剂对OBS的吸附容量。


*q_t_* =(*C*_0_-*C_t_* )*V*/*m* (1)

其中，*q_t_* （mg/g）是OBS在时间*t*（min）时的吸附容量，*C*_0_与*C_t_* （mg/L）分别是OBS溶液的初始浓度和时间*t*（min）时的剩余浓度，*V*（mL）是OBS溶液的体积，*m*（mg）是吸附剂的质量。

## 2 结果与讨论

### 2.1 材料的表征

如图2所示，U-TFA-10 M和UN-TFA-10 M均未呈现良好的八面体形貌并且晶体之间存在一定的重叠，U-TFA-10 M的尺寸比UN-TFA-10 M略大，该现象与文献［^31^］报道相似。羧酸基团通过Zr-O簇进行配位，当配位点被TFA占据后晶体无法向该方向继续生长以获得完美的三维拓扑结构，因此材料形貌不够规整。通过FT-IR图谱分析了所合成材料的化学结构，如图3a所示，在556 cm^-1^和482 cm^-1^处出现的吸收峰来自Zr-O配位，U-TFA-10 M、UN-TFA-10 M在1 212 cm^-1^处产生了新的特征峰，这源于C-F键的拉伸振动，证实了TFA成功引入^［31-33］^。从XPS谱图（图3b）能够看到C、O、F和Zr元素的存在，与U-TFA-10 M相比，UN-TFA-10 M含有明显的N元素。研究了不同浓度TFA调控的U-TFA-*n* M与UN-TFA-*n* M系列材料的晶体结构。如图3c、3d所示，经TFA调节的U-TFA-*n* M与UN-TFA-*n* M在2*θ*为5.5°、7.4°等特征衍射峰位置与原始UiO-66、UiO-66-NH_2_保持高度一致，说明使用TFA构建缺陷的同时未改变材料的三维拓扑结构。经TFA调控的UiO-66系列材料在2*θ*为3°~5°范围内出现一个新的宽峰，这是由于U-TFA-*n* M、UN-TFA-*n* M存在配体缺失缺陷^［34］^。以上结果说明使用三氟乙酸作为调节剂在U-TFA-10 M和UN-TFA-10 M中成功制造了缺陷。

**图2:**
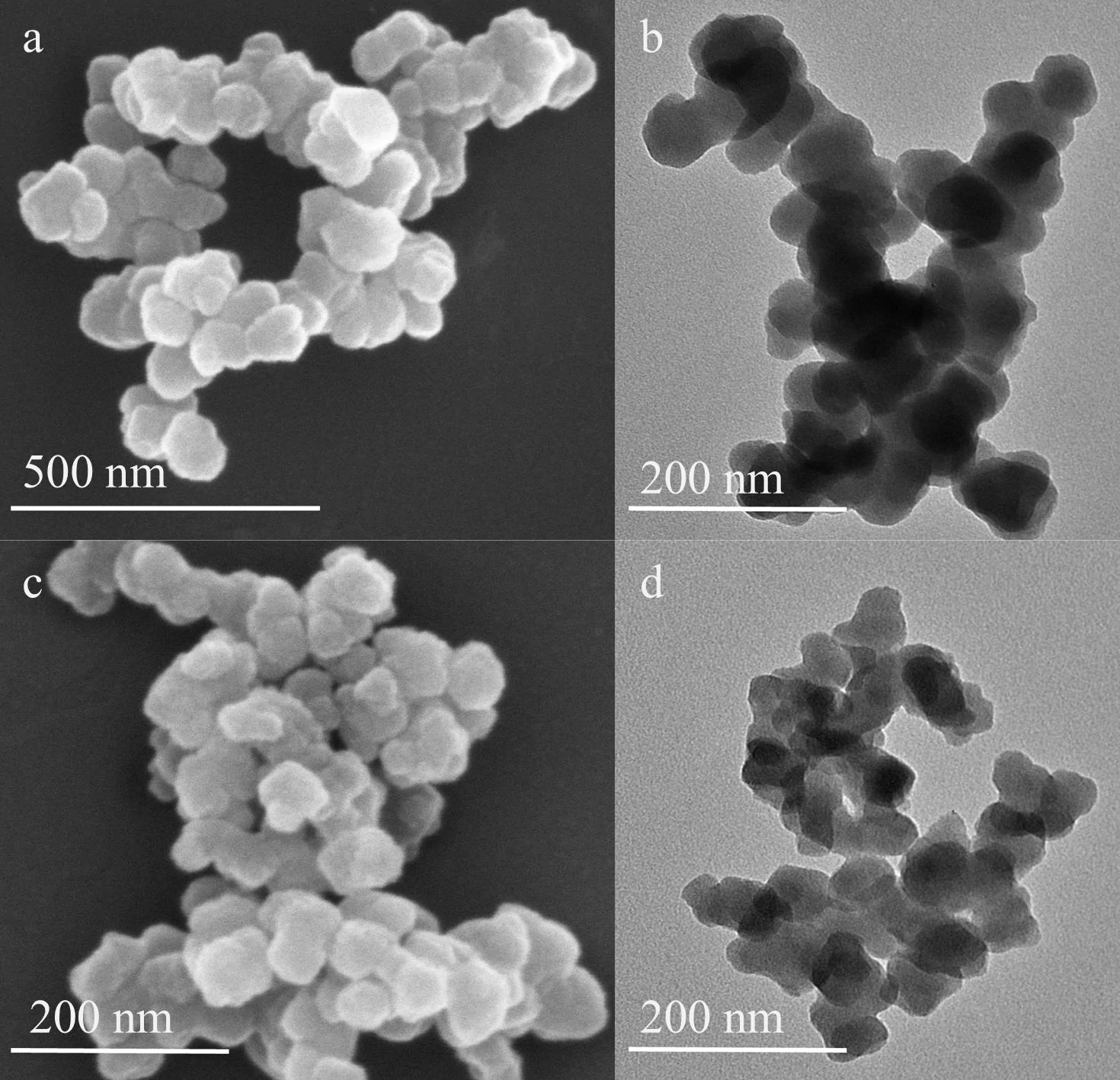
U-TFA-10 M的（a）SEM图和（b）TEM图； UN-TFA-10 M的（c）SEM图和（d）TEM图

**图3:**
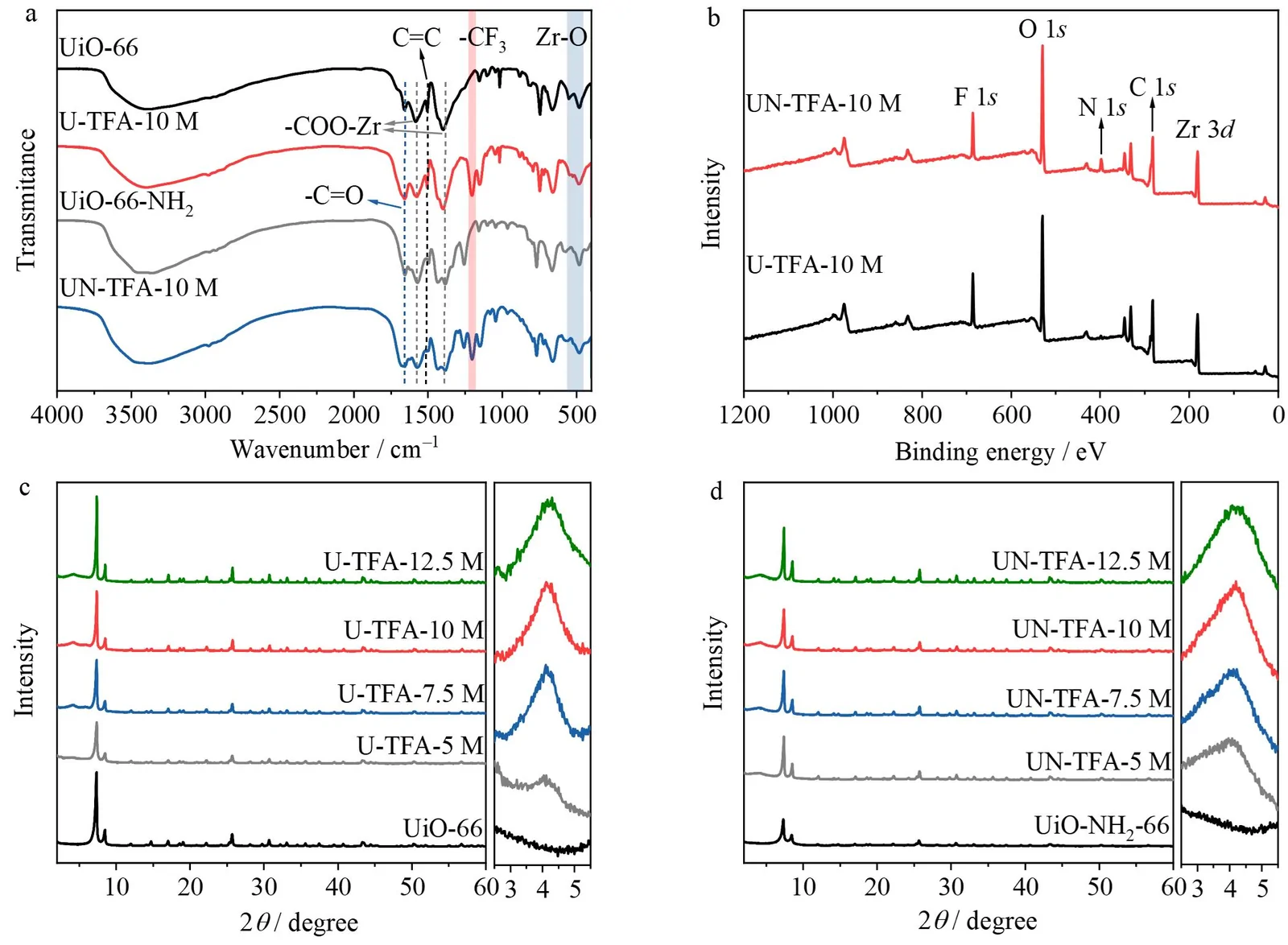
（a）UiO-66系列材料的FT-IR图；（b）U-TFA-10 M、UN-TFA-10 M的XPS谱图；不同浓度TFA调控（c）UiO-66、（d）UiO-66-NH_2_的XRD谱图

### 2.2 吸附条件的优化

使用相同浓度、不同酸度和碳链长度的单羧酸（AA、PA、TFA）调控的吸附剂对OBS进行吸附，结果如图4a所示，U-TFA-10 M和UN-TFA-10 M的吸附效果显著优于其他吸附剂，这是由于经过TFA调控，成功引入-CF_3_官能团，通过氟-氟相互作用和疏水相互作用提高对OBS的吸附性能，且TFA酸性强，能产生更多配位缺陷。未经调控的UiO-66、UiO-66-NH_2_对OBS几乎没有吸附效果，与文献［^29^］报道研究结果一致。对比不同浓度TFA调节的U-TFA-*n* M与UN-TFA-*n* M的吸附能力，结果表明（图4b），当调节剂TFA浓度为10 mol/L时，吸附效果最好。适当浓度的TFA通过竞争配位在UiO-66、UiO-66-NH_2_中引入缺陷，增加孔隙率和活性位点，但过高浓度可能破坏框架结构，造成吸附能力的下降。用体积可忽略不计的0.1 mol/L HCl和0.1 mol/L NaOH调节OBS分析物溶液的pH值，考察了吸附剂在pH值为3~9范围内对OBS的吸附情况。如图4c所示，在酸性条件下，U-TFA-10 M和UN-TFA-10 M对OBS有较高的吸附容量，但随着溶液pH的升高，吸附容量显著下降。水环境中存在的大量无机阴离子可能会与OBS竞争吸附位点，在不同浓度的氯化钠溶液中，探究了离子强度对吸附性能的影响。如图4d，当Cl^-^质量浓度从0上升至100 mg/L时，U-TFA-10 M、UN-TFA-10 M的吸附容量略有上升或下降，无明显的线性相关性，离子强度对OBS吸附效率的影响可以忽略不计。

**图4:**
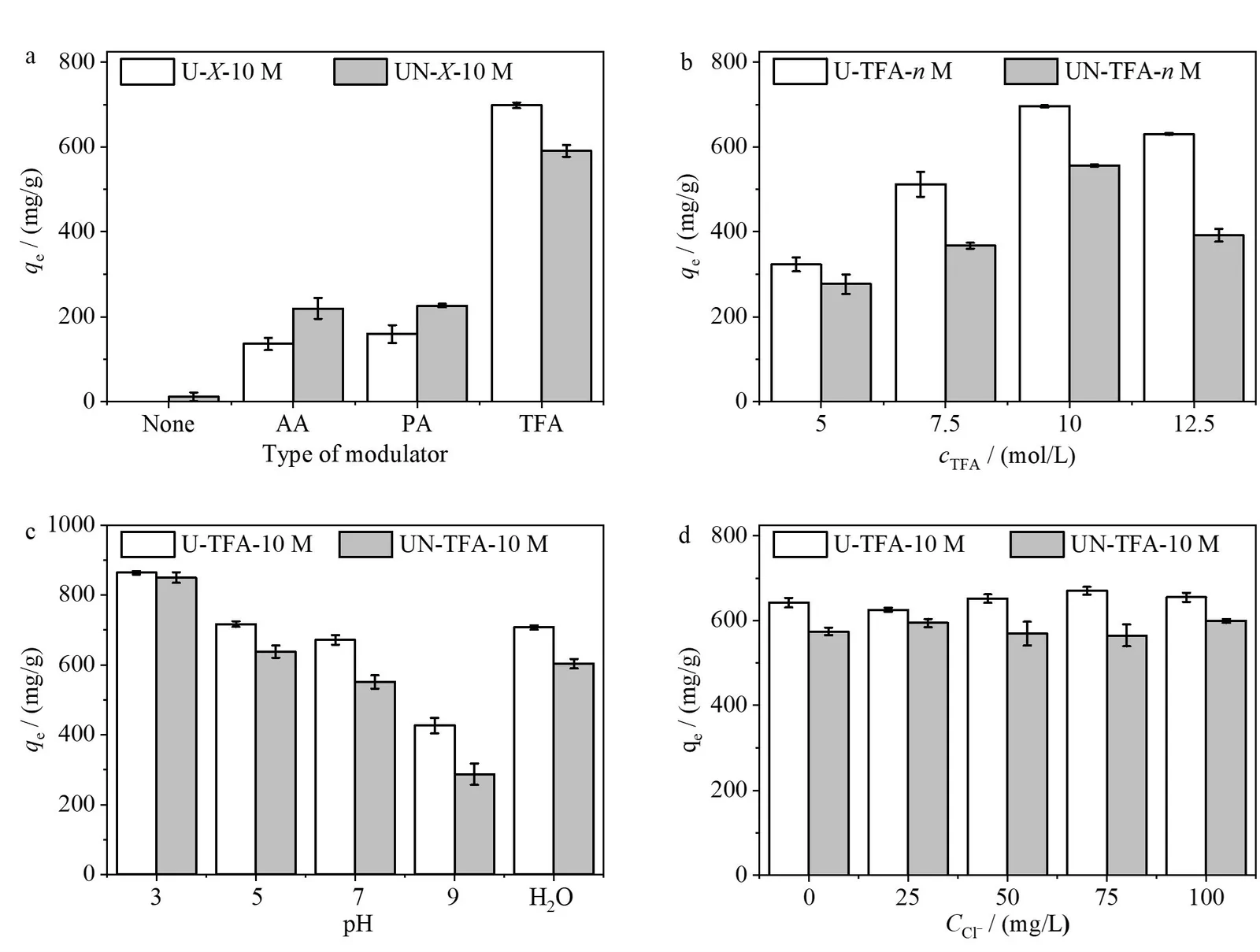
（a）调节剂种类、（b）TFA浓度、（c）pH和（d）Cl **^-^** 质量浓度对UiO-66系列材料吸附OBS的影响

### 2.3 吸附等温线和吸附动力学

通过在298 K下改变OBS的初始浓度进行吸附等温线研究，探究吸附剂与OBS之间的相互作用及两种吸附剂的表面特性。吸附等温线结果（图5a）表明，U-TFA-10 M和UN-TFA-10 M对OBS的平衡吸附容量随初始浓度的增加先快速上升后趋于平缓。通过模型拟合，Langmuir模型（*R* ^2^>0.999 5）相对于Freundlich模型（*R* ^2^<0.973 0）具有更好的线性关系（图5b、5c），说明OBS在两种吸附剂表面的吸附行为符合单分子层吸附特征，不存在OBS分子之间的相互作用^［35］^。根据Langmuir模型计算得到U-TFA-10 M和UN-TFA-10 M的理论最大吸附容量分别是684.9 mg/g和602.4 mg/g，表现出优异的吸附性能。

**图5:**
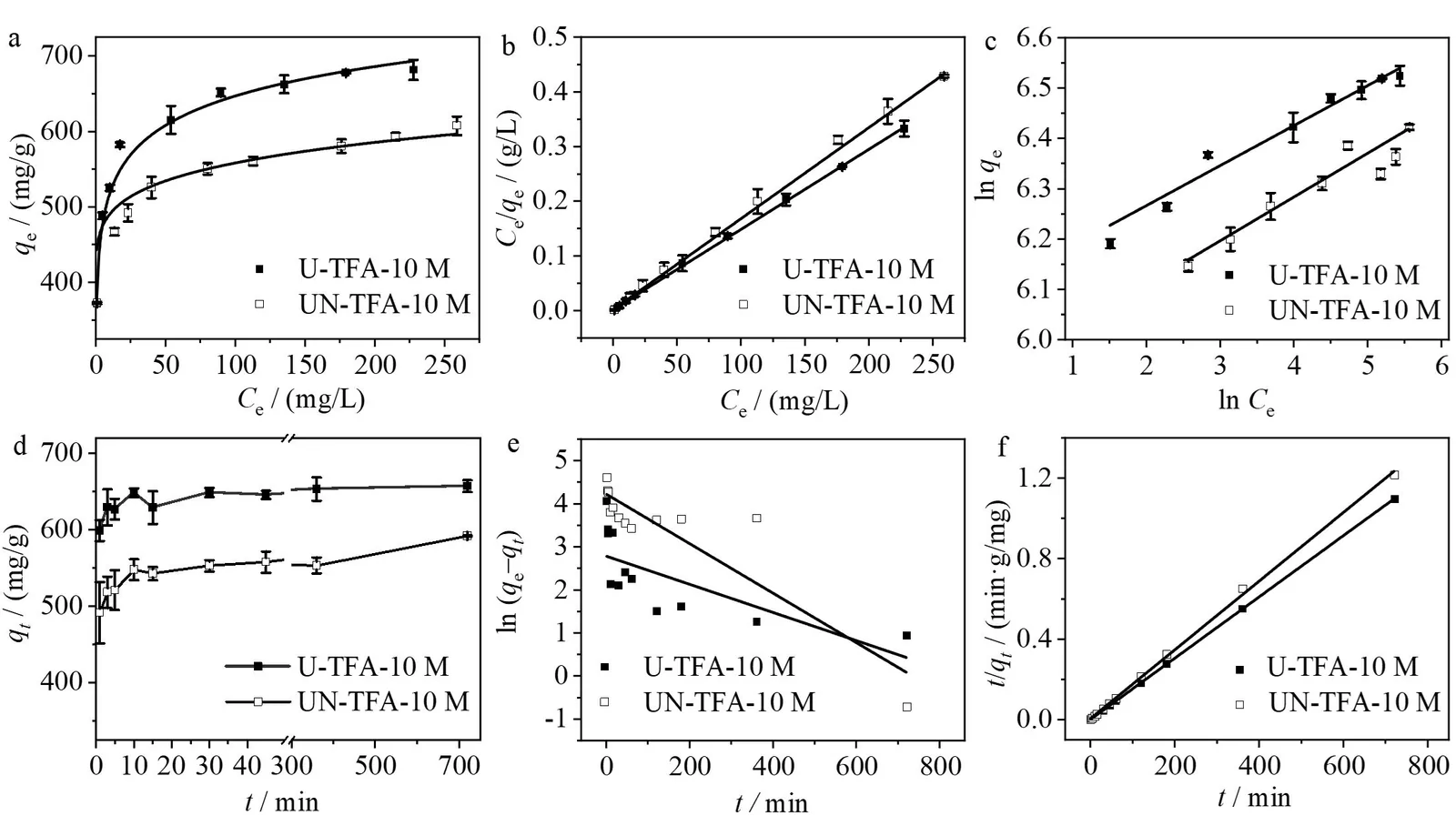
OBS在U-TFA-10 M和UN-TFA-10 M上的（a）吸附等温线、（b）Langmuir模型拟合曲线、（c）Freundlich模型拟合曲线、（d）吸附动力学曲线、（e）伪一级动力学模型拟合曲线和（f）伪二级动力学模型拟合曲线

通过改变吸附剂与OBS溶液之间的接触时间，研究了U-TFA-10 M和UN-TFA-10 M的吸附动力学行为。如图5d所示，两种吸附剂在10 min内对OBS的去除率达90%以上，表明其具有快速的吸附动力学特性。随后，吸附速率逐渐降低，并在30 min内达到平衡。这一现象主要归因于在初始阶段材料表面存在大量未被占据的活性位点，随着吸附的进行，剩余吸附位点逐渐减少，最终达到动态平衡。采用伪一级动力学和伪二级动力学模型进行拟合分析，结果表明（如图5e、5f），伪一级动力学模型拟合偏差较大，两种吸附剂的吸附更符合伪二级动力学模型，其线性相关系数更高，说明OBS在U-TFA-10 M或UN-TFA-10 M上的吸附行为受化学吸附机制主导。

### 2.4 重复与再生能力

重复与再生能力是评价吸附剂应用性能的重要参数。通过吸附-解吸-再生实验探究了U-TFA-10 M、UN-TFA-10 M的再生性能，如图6a，经过一次再生循环后，再生材料吸附容量有所降低；但是相较于未再生的商用吸附剂阴离子交换树脂（IRA-900）、活性炭来说，再生材料（Re-U：再生U-TFA-10 M，Re-UN：再生UN-TFA-10 M）仍然具有更优的吸附效果（图6b），在实际应用方面具有一定前景。对比发现，UN-TFA-10 M的再生材料相较于原始材料吸附性能变化相对较小，说明其再生性能相对更好。

**图6:**
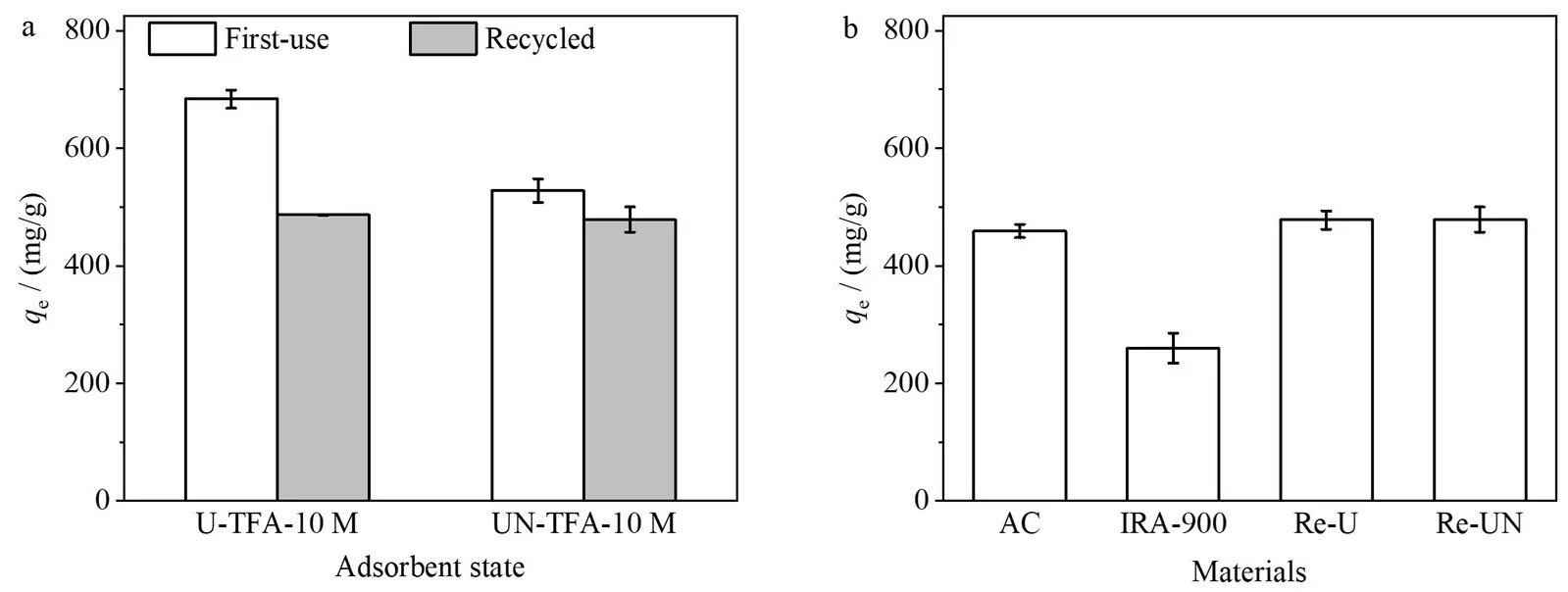
（a）U-TFA-10 M和UN-TFA-10 M的原始材料与再生材料吸附性能对比；（b）再生U-TFA-10 M和UN-TFA-10 M与商用吸附剂吸附性能对比

### 2.5 吸附机理

U-TFA-10 M和UN-TFA-10 M对新型污染物OBS表现出优越的吸附性能，基于Freundlich模型计算的非线性指标*n*值远高于1，说明吸附过程中涉及多种吸附机制。综合表征测试结果、吸附实验以及分析物的结构特性，分析了TFA调控缺陷UiO-66系列材料对OBS的可能存在的吸附机理：孔径匹配、静电相互作用、酸碱相互作用、氟-氟相互作用和疏水相互作用。

N_2_吸附-脱附实验表明TFA调控后材料具有高比表面积和孔径，U-TFA-10 M和UN-TFA-10 M的孔径分别为1.73 nm、1.93 nm，OBS的分子长度为1.26 nm^［36］^，而UiO-66、UiO-66-NH_2_对OBS基本没有吸附效果，说明了可能存在孔径匹配作用。XRD谱图（图3c、3d）中U-TFA-10 M和UN-TFA-10 M在2*θ*为3°~5°范围内宽峰的出现表明TFA成功制造了缺陷。吸附完成后，XRD谱图（图7a）在2*θ*为3°~5°范围内的峰消失，说明OBS与Zr进行了结合，吸附过程可能涉及酸碱相互作用。不同pH下的吸附实验（图4c）表明，在低pH条件下，吸附剂表面高度质子化带正电荷，吸附效果显著，随着pH升高，质子化程度降低，吸附量出现下降，这说明吸附过程中存在静电相互作用。在低pH条件下，UN-TFA-10 M与U-TFA-10 M的吸附差距缩小，这是由于UN-TFA-10 M的-NH_2_在低pH下质子化为-NH_3_^+^，静电作用加强。虽然pH为3时吸附效果最好，但如图7b所示，此时Zeta电位不是最高值，说明吸附过程受其他因素影响。如图4a所示，U-TFA-10 M和UN-TFA-10 M的吸附效果显著优于其他单羧酸调控的吸附剂，表明引入的-CF_3_官能团与OBS的全氟烷基疏水链之间存在氟-氟相互作用和疏水相互作用。通过选择性吸附实验（图7c），U-TFA-10 M和UN-TFA-10 M对OBS的吸附量显著高于其他污染物（BA、2-FBA、SDBS），这可能是因为OBS分子尺寸与材料孔径匹配度更高，也说明了孔径匹配作用的存在。U-TFA-10 M和UN-TFA-10 M对SDBS和OBS吸附效果相对更好，这是因为强酸性磺酸基团更易与质子化吸附剂表面结合，也说明了静电作用的存在。2-FBA吸附效果略优于BA，说明可能通过氟-氟相互作用进行吸附。

**图7:**
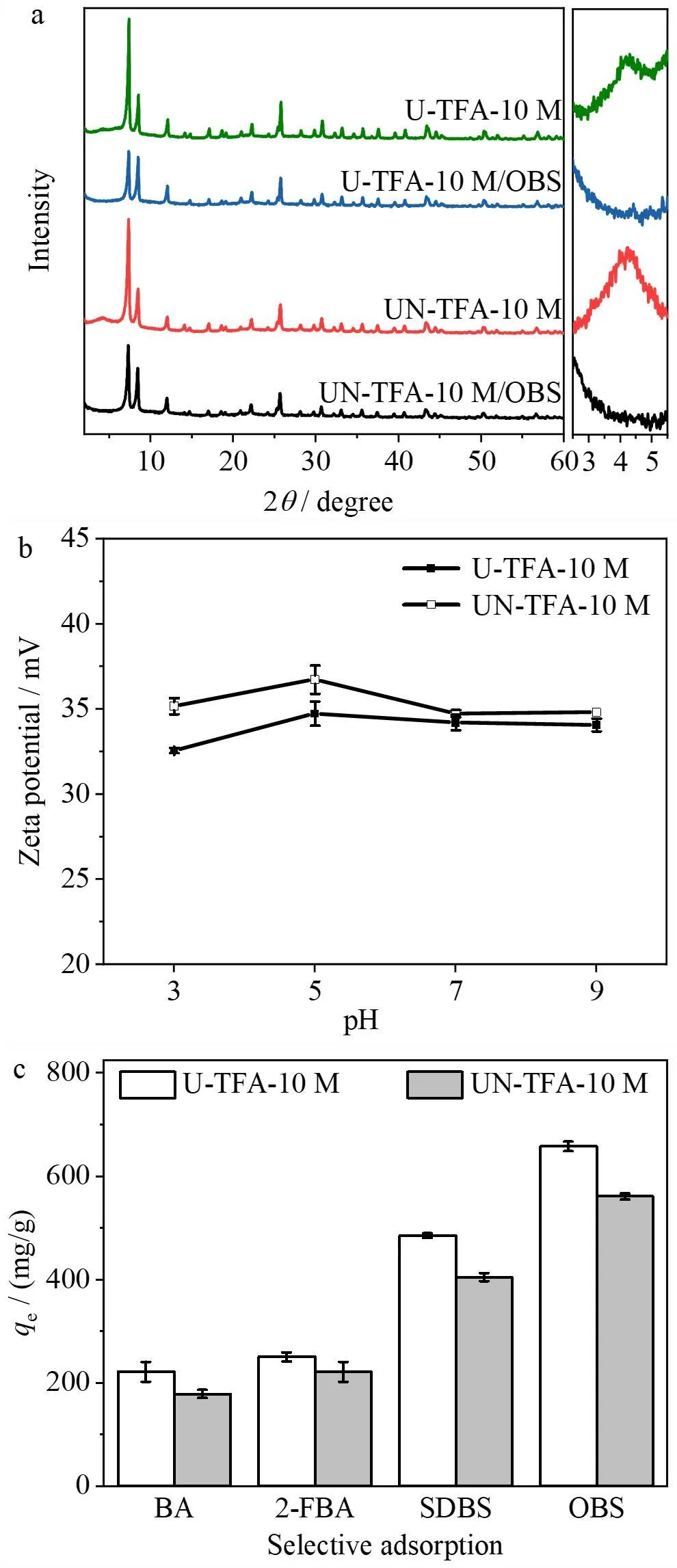
U-TFA-10 M和UN-TFA-10 M（a）吸附OBS前后的XRD图、（b）Zeta电位图和（c）对不同分析物的吸附

### 2.6 与其他吸附剂对比

由于OBS是PFOS的新型替代品，目前针对OBS的研究相对较少，相较于其他已报道的OBS的吸附剂（表1），U-TFA-10 M与UN-TFA-10 M具有更高的吸附容量和更快的吸附平衡时间。

**表1:** 本研究开发的吸附剂与其他吸附剂对OBS的吸附效果比较

Adsorbents	*q*_m，exp_/（mg/g）	BET/（m^2^/g）	*t/*h	Ref.
R2-GAC	382.9	1705.0	72	［^5^］
Fe_3_O_4_/rGO	241.8	95.5	120	［^36^］
CTF2/Fe_3_O_4_	783.8	852.4	3	［^37^］
UiO-66-F_2_	215.6	593.0	24	［^28^］
UiO-66-F_4_	451.2	814.0	24	［^28^］
U-TFA-10 M	668.5	1819.0	0.5	this work
UN-TFA-10 M	592.7	1450.0	0.5	this work

*q*_m，exp_：experimental maximum adsorption capacity； R2-GAC： reactivated granular-activated carbon； Fe_3_O_4_/rGO： magnetic graphene-based Fe_3_O_4_ composites； CTF2/Fe_3_O_4_： magnetic covalent organic frameworks； UiO-66-F_2_/UiO-66-F_4_： metal-organic frameworks.

## 3 结论

本工作采用溶剂热法制备了由三氟乙酸调控的U-TFA-10 M和UN-TFA-10 M两种缺陷MOFs，用于全氟化合物OBS的吸附。三氟乙酸作为调节剂，其羧酸基团通过Zr-O簇与原配体竞争配位，实现了对UiO-66的缺陷调控，提供了更大的比表面积、孔体积以及更多活性吸附位点。引入的-CF_3_官能团使材料可以通过孔径匹配、静电相互作用、酸碱相互作用、氟-氟相互作用和疏水相互作用实现对OBS的高效吸附，具有优异的选择性、快速的吸附速率和较强的吸附能力，合成方法操作简便，在吸附去除OBS的应用中具有巨大的潜力。
